# Association of Clinical, Radiological, and Procedural Characteristics with NIHSS at Discharge and 90-Day Modified Rankin Scale Outcomes in Individuals with Stroke Treated with Mechanical Thrombectomy

**DOI:** 10.3390/diagnostics16142137

**Published:** 2026-07-08

**Authors:** Damljan Bogićević, Filip Vitošević, Marjana Vukićević, Anđela Gogić, Vojin Kovačević, Aleksandar Ćirović

**Affiliations:** 1 Special Hospital for Cerebrovascular Diseases “Sveti Sava”, 11000 Belgrade, Serbia; damljanbogicevic@yahoo.com (D.B.); filipvitosevic@gmail.com (F.V.);; 2 Faculty of Medical Sciences, University of Kragujevac, 34000 Kragujevac, Serbia; 3 Faculty of Medicine, University of Belgrade, 11000 Belgrade, Serbia; 4 Department of Medical Statistic, Faculty of Medical Sciences, University of Kragujevac, 34000 Kragujevac, Serbia; 5 Department of Surgery, Faculty of Medical Sciences, University of Kragujevac, 34000 Kragujevac, Serbia; vojinkg@gmail.com; 6 Faculty of Medicine, Institute of Anatomy, University of Belgrade, Dr Subotica 4/2, 11000 Belgrade, Serbia

**Keywords:** mechanical thrombectomy, NIHSS at discharge, 90-day mRS, ischemic stroke

## Abstract

**Background:** This study aimed to evaluate associations between baseline clinical status, radiological findings, prior medical history, procedural characteristics, and recanalization success with neurological outcome at discharge (NIHSS) and functional status at 90 days (mRS). **Methods:** This single-center retrospective study included 100 consecutive patients with acute ischemic stroke treated with MT within 24 h of symptom onset. Clinical data, CT perfusion parameters, comorbidities, prior therapies, and detailed procedural characteristics (including anesthesia type, recanalization grade, blood pressure variability, hemorrhagic transformation, and procedure duration) were analyzed. Nonparametric tests (Mann–Whitney U, Kruskal–Wallis) and Spearman’s rank correlation were applied. Statistical significance was set at *p* < 0.05. **Results:** Early ischemic signs on baseline imaging were associated with higher NIHSS scores at discharge and worse 90-day mRS (*p* < 0.05). Patients undergoing CT perfusion imaging had higher discharge NIHSS. Admission NIHSS showed a moderate positive correlation with discharge NIHSS (rho = 0.367, *p* = 0.003). Strong correlations were observed between NIHSS at 24 h and discharge NIHSS (rho = 0.802, *p* < 0.001), as well as between 24 h NIHSS and 90-day mRS (rho = 0.842, *p* < 0.001). Successful recanalization was significantly associated with long-term outcomes, with mTICI 3 associated with the lowest 90-day mRS scores (*p* = 0.003). Intraprocedural blood pressure variability, hemorrhagic transformation, infections, and in-hospital complications were all linked to higher 90-day mRS values. Prolonged procedure duration showed a weak but significant correlation with worse 90-day outcome (rho = 0.232, *p* = 0.020). In contrast, prior comorbidities and pre-stroke therapy were not significantly associated with outcomes. A multivariable binary logistic regression analysis identified only the NIHSS score at 24 h remained as an independent predictor of favorable functional outcome at 90 days. **Conclusions:** Baseline stroke severity, early neurological evolution, successful reperfusion, procedural hemodynamic stability, and prevention of in-hospital complications are strongly associated with both early neurological recovery and long-term functional outcome after mechanical thrombectomy.

## 1. Introduction

Ischemic stroke remains one of the leading causes of death and long-term disability worldwide. According to the Global Burden of Disease (GBD) 2021 study, stroke was the third leading cause of death globally, responsible for 7.3 million deaths (10.7% of all deaths), ranking behind only ischemic heart disease and COVID-19, and the fourth leading cause of disability-adjusted life years (DALYs), accounting for 160.5 million DALYs [1]. Ischemic stroke is the most common subtype, comprising approximately 65.3% of all incident strokes [1]. In 2021, an estimated 7.8 million new ischemic strokes occurred worldwide [2], causing approximately 3.59 million deaths [2,3]. Based on Global Burden of Disease Study 2021 projections, the global number of incident ischemic strokes is projected to increase by 40.5% between 2021 and 2050 [4]. Survivors of ischemic stroke frequently experience long-term disability, which is often accompanied by post-stroke depression, anxiety, apathy, and fatigue [5]. These individuals exhibit significantly reduced quality of life and impose a considerable burden on their family members and caregivers. In addition, the treatment and long-term care of stroke survivors are associated with substantial healthcare costs [6]. Thus, there is a substantial space for further evaluation of factors that may influence outcomes in the individuals once a stroke has appeared. Mechanical thrombectomy is standard procedure for the treatment of various neurological conditions such as brain aneurisms [7] and acute ischemic stroke and should be performed as soon as possible after symptom onset, particularly when large blood vessels are occluded [8]. Lambrinos et al. in their systematic review demonstrated that although there were no differences in terms of mortality between individuals who were medically treated and those who were treated with mechanical thrombectomy, those patients who underwent mechanical thrombectomy had significantly improved functional independence measured by a modified Rankin score [9].

The National Institutes of Health Stroke Scale (NIHSS) is a standardized clinical tool consisting of 10 items, designed to quantify the severity of neurological impairment in patients with acute stroke. Scores range from 0, indicating no detectable deficit, to 42, reflecting profound neurological dysfunction [10]. The NIHSS is widely used to support clinical decision-making, including eligibility for reperfusion therapies such as thrombolysis and thrombectomy, as well as to estimate prognosis and track neurological recovery over time. Increasing scores correspond to greater stroke severity, commonly classified as minor (1–4), moderate (5–15), and severe (16–42), with defined score ranges also providing insight into anticipated rehabilitation requirements.

The modified Rankin Scale (mRS) assessed at 90 days is a widely accepted measure of functional outcome after stroke. It evaluates the degree of disability and dependence in daily activities, rather than acute neurological impairment. Scores range from 0 (no symptoms) to 6 (death), with lower scores indicating better functional recovery. In clinical research, an mRS score of 0–2 at 90 days is commonly defined as a favorable outcome, while scores of 3–6 reflect poor functional prognosis. Studies examining the associations between clinical, radiological, and biochemical factors and both early neurological deterioration and 90-day functional outcome have largely been conducted in cohorts treated with intravenous thrombolysis. Consequently, the impact of clinical, radiological, and procedural factors during mechanical thrombectomy on these functional outcome measures remains insufficiently investigated.

The aim of this paper is to evaluate relationships between baseline clinical status, radiological findings, prior medical history, treatment characteristics, and procedural outcomes with NIHSS at discharge and 90-day mRS.

## 2. Methods

This study was designed as a single-center retrospective study. For this study, we enrolled 100 consecutive patients diagnosed with ischemic stroke (mean age 72.14 ± 11.75 years, range 33–93) who were treated at the Special Hospital for Cerebrovascular Diseases. Of the total, 37 were male (mean age 68.89 ± 14.35) and 63 were female (mean age 74.05 ± 9.54).

Inclusion criteria. Patients were included if they met all of the following criteria: (1) LVO confirmed on CT angiography involving the internal carotid artery (ICA, including the terminal segment), a tandem occlusion, or the M1, M2, or M3 segment of the middle cerebral artery (MCA); (2) MT initiated within 24 h from symptom onset to arterial puncture, with onset defined as the time the patient was last known to be well in the case of wake-up strokes; (3) penumbra/core mismatch ≥ 1.8 on CT perfusion; (4) ischemic core volume ≤ 100 mL; (5) ASPECTS ≥ 3 on non-contrast CT; (6) pre-stroke mRS 0–2. Bridging intravenous thrombolysis prior to MT was permitted and was administered in six patients. The median time from stroke-center arrival to arterial puncture was 110 min, and to recanalization 160 min.

Exclusion criteria. Patients were excluded for (1) intracranial hemorrhage on non-contrast CT; (2) absence of LVO; (3) occlusion of the M4 segment or more distal branches, isolated anterior cerebral artery occlusion, or occlusion within the vertebrobasilar territory; (4) age below 18 years; (5) terminal-stage malignancy; (6) severe renal insufficiency; (7) platelet count < 40 × 10^9^/L. Patients in whom spontaneous recanalization was identified during the procedure were also excluded. No patients were excluded because of missing or incomplete data; complete baseline (clinical and radiological), procedural, and 90-day follow-up data were available for all 100 consecutive patients.

All included patients underwent mechanical endovascular thrombectomy within 24 h of symptom onset. All procedures were performed under one of three anesthetic modalities: local anesthesia (LA), conscious sedation (SED), or general endotracheal anesthesia (OETA). Intraprocedural blood pressure variability was not quantified as a measure of statistical dispersion (such as the standard deviation or coefficient of variation); instead, it was recorded as a binary variable, defined a priori as a systolic arterial pressure variation greater than 30 mmHg and/or a diastolic arterial pressure variation greater than 20 mmHg during the procedure. According to protocol, cranial CT was performed before and after the procedure. Information on prior medications and medical history was obtained from the hospital records. Infectious complications occurring during hospitalization were ascertained from the hospital records; all infections recorded in this cohort were pneumonia. Cranial CT parameters included in the study were: CT perfusion core volume (mL), CT perfusion penumbra volume (mL), penumbra/core ratio, penumbra/core mismatch ratio, occlusion type, and other CT perfusion metrics. The study was approved by the Ethical Committee of the Special Hospital for Cerebrovascular Diseases (approval no. 03/5708).

## 3. Statistical Analysis

An a priori power analysis was performed using G*Power version 3.1.9.4. Assuming a medium effect size (f^2^ = 0.15), a significance level of α = 0.05, statistical power of 80%, and four predictors corresponding to the final multivariable regression models, the minimum required sample size was estimated to be 85 participants. As the present study included 100 patients, the achieved sample size exceeded the required minimum, indicating adequate statistical power for the planned regression analyses. To minimize the risk of model overfitting, the number of predictors included in the final models was restricted according to the number of outcome events, results of the univariate analyses and clinical relevance. ASPECTS (≥3), ischemic core volume (≤100 mL), and the penumbra/core mismatch ratio (≥1.8) were applied as eligibility criteria, so that the corresponding variables had a restricted range within the cohort (for example, ASPECTS: median 9, range 5–10); consistent with this, none of the quantitative CT perfusion parameters showed a significant univariate association with NIHSS at discharge or 90-day mRS (Table 1, all *p* > 0.05). These imaging measures were therefore not entered as candidate predictors, whereas the binary indicator of whether CT perfusion imaging was performed was retained as a candidate, functioning as a surrogate of baseline clinical severity rather than a direct perfusion measurement. Nonparametric analyses were used throughout: group comparisons were performed with the Mann–Whitney U test for two-group comparisons and the Kruskal–Wallis test (reported with χ^2^) for multi-group comparisons, with results presented as median (IQR) to assess differences in NIHSS at discharge and 90-day mRS across clinical (e.g., wake-up stroke, early CT signs, infections), radiological (CT perfusion, hyperdense artery, occlusion site) and procedural variables (anesthesia type, first angiogram mTICI, final recanalization, blood-pressure variability, hemorrhagic transformation); post hoc Bonferroni adjustments were applied for multiple pairwise comparisons where noted.

Associations between continuous or ordinal variables (clinical, hemodynamic, CT perfusion volumes, procedural times, number of passes, NIHSS at different timepoints) and outcomes were assessed using Spearman’s rank correlation (Spearman’s rho) with corresponding *p*-values. Descriptive group counts and medians were reported, and statistical significance was taken at *p* < 0.05. Univariate analyses were performed primarily for descriptive and exploratory purposes and to identify candidate variables for multivariable modeling. Bonferroni adjustment was applied only to post hoc pairwise comparisons following significant omnibus tests involving multiple group comparisons. No global adjustment for multiple testing was applied across the exploratory univariate analyses; accordingly, the principal inferences of the study are derived from the multivariable regression models rather than from individual univariate comparisons.

To identify independent predictors of favorable functional outcome at 90 days, a multivariable binary logistic regression analysis was performed. Favorable outcome was defined as a modified Rankin Scale (mRS) score of 0–2, whereas mRS scores of 3–6 were considered unfavorable outcomes. Model fit was evaluated using the likelihood ratio χ^2^ test, the Hosmer–Lemeshow goodness-of-fit test, Nagelkerke’s R^2^ and overall classification accuracy. Multicollinearity was assessed using variance inflation factors (VIF) and tolerance statistics. Odds ratios (ORs) with 95% confidence intervals (95% CIs) were reported.

An ordinal logistic regression analysis was performed to identify independent predictors of neurological status at discharge. NIHSS severity at discharge was categorized into three ordered levels (minor, moderate, and severe neurological deficit). The proportional odds assumption was assessed using the Test of Parallel Lines. Model fit was evaluated using the likelihood ratio χ^2^ test, Pearson and Deviance goodness-of-fit statistics and Nagelkerke’s pseudo-R^2^. Multicollinearity diagnostics were performed before fitting the final model, and ORs with 95% CIs were reported.

## 4. Results

Based on the results of the Mann–Whitney U test, no statistically significant difference was found in NIHSS scores at discharge between male and female patients (*p* > 0.05). However, a statistically significant difference was observed in the 90-day modified mRS scores (*p* < 0.05), with women having a higher median score (Median = 5.00) compared to men (Median = 3.00).

A moderate, positive, and statistically significant correlation was found between patient age and 90-day mRS scores (Spearman’s rho = 0.319; *p* = 0.001), as well as between age and NIHSS scores at discharge (Spearman’s rho = 0.300; *p* = 0.018).

Based on the results of the Kruskal–Wallis test, no statistically significant differences were found in either NIHSS scores at discharge or 90-day mRS scores (*p* > 0.05) among groups defined by different smoking status.

Based on the results of the Mann–Whitney and Kruskal–Wallis tests, a statistically significant difference in NIHSS scores at discharge was identified with respect to the presence of early signs of stroke, as well as with respect to the use of CT perfusion imaging (*p* < 0.05). Patients with early signs of stroke had higher NIHSS scores at discharge (Median = 8.00) compared with those in whom early signs were not present (Median = 4.00). In addition, higher NIHSS scores were also observed in patients who underwent CT perfusion imaging (Median = 7.00) compared with patients without perfusion diagnostics (Median = 3.00). A statistically significant difference in the Rankin score at 90 days was also found in relation to the presence of early signs of stroke (*p* < 0.05), with patients exhibiting early signs possessing higher Rankin scores (Median = 5.00) compared with patients without early signs (Median = 2.00) (Table 2).

A moderate, positive, and statistically significant correlation was identified between NIHSS scores at admission and NIHSS scores at discharge (Spearman’s rho = 0.367; *p* = 0.003). For the remaining analyzed parameters, including systolic and diastolic blood pressure, as well as CT perfusion parameters, no statistically significant correlation was found with either NIHSS scores at discharge or Rankin scores at 90 days (*p* > 0.05) (Table 1).

Table 3 presents the association between characteristics of prior medical history and previously applied therapy with NIHSS scores at discharge and Rankin scores at 90 days. Based on the results of the Mann–Whitney U test, no statistically significant differences were found, either in NIHSS scores at discharge or in Rankin scores at 90 days, for any of the examined variables, including previous mRS score, the presence of hypertension, atrial fibrillation, cardiomyopathy, diabetes mellitus, hyperlipidemia, as well as prior antiplatelet, anticoagulant, and statin therapy (*p* > 0.05).

Based on the results of the Mann–Whitney and Kruskal–Wallis tests, a statistically significant difference in Rankin scores at 90 days was found with respect to the degree of recanalization (*p* < 0.05), with patients achieving mTICI = 3 having the lowest Rankin score at 90 days (Median = 2.50). In addition, a statistically significant difference in Rankin scores at 90 days was observed with respect to the presence of arterial blood pressure variations during the procedure (*p* < 0.05), whereby patients with pronounced variations had higher Rankin scores (Median = 6.00) compared with patients without significant variations (Median = 3.00). A statistically significant difference in Rankin scores at 90 days was also noted in relation to the presence of hemorrhagic transformation (*p* < 0.05), with patients who developed hemorrhagic transformation having significantly worse scores (Median = 6.00) compared with those without transformation (Median = 3.00) (Table 4).

First angiogram analysis revealed that lower mTICI score was linked with lower NIHSS at discharge (*p* = 0.049). A strong, positive and statistically significant correlation was found between the NIHSS score after completion of the procedure and the mRS score at 90 days (Spearman’s rho = 0.502; *p* = 0.000), as well as a strong, positive, and statistically significant correlation between the NIHSS score at 24 h and the mRS score at 90 days (Spearman’s rho = 0.842; *p* = 0.000). A strong, positive and statistically significant correlation was also recorded between the NIHSS score at 24 h and the NIHSS score at discharge (Spearman’s rho = 0.802; *p* = 0.000), and between the NIHSS score after completion of the procedure and the NIHSS score at discharge (Spearman’s rho = 0.661; *p* = 0.000). For other analyzed parameters (NIHSS before start of mechanical thrombectomy, number of passes), no statistically significant correlation with mRS at 90 days was found (*p* > 0.05) (Table 5).

A weak, positive, and statistically significant correlation was found between the duration of mechanical thrombectomy (time from puncture to recanalization/completion of the intervention) and the RANKIN score after 90 days (Spearman’s rho = 0.232; *p* = 0.020). For the other analyzed time parameters, no statistically significant correlation was observed with either the NIHSS score at discharge or the RANKIN score after 90 days (*p* > 0.05) (Table 6).

A weak, positive correlation was observed between the number of days of hospitalization and the NIHSS score at discharge (Spearman’s rho = 0.247; *p* = 0.053), but it did not reach statistical significance. Likewise, no statistically significant correlation was found between the length of hospital stay and the RANKIN score after 90 days (Spearman’s rho = −0.130; *p* = 0.196).

Based on the results of the Mann–Whitney and Kruskal–Wallis tests, a statistically significant difference in NIHSS scores at discharge was found with respect to the presence of infections (*p* < 0.05), with patients who developed infections having a higher median NIHSS score (Median = 10.00) compared with those without infections (Median = 6.00). A statistically significant difference in NIHSS scores was also observed in relation to the RANKIN score at discharge (*p* < 0.05), showing a gradual increase in median NIHSS values with increasing levels of disability.

Regarding the RANKIN score after 90 days, statistically significant differences were identified in relation to several parameters, including the presence of dissection, the occurrence of complications during hospitalization, infections, and the RANKIN score at discharge. Patients with complications and infections had higher RANKIN scores. In addition, a higher RANKIN score at discharge was associated with a significantly worse RANKIN score after 90 days, with the median increasing as the degree of disability increased (Table 7).

A multivariable binary logistic regression analysis was performed to identify independent predictors of favorable functional outcome at 90 days (mRS 0–2). The model was statistically significant and demonstrated good overall performance (Table 8). After adjustment for all variables included in the model, only the NIHSS score at 24 h remained an independent predictor of favorable functional outcome at 90 days (OR = 0.545, 95% CI 0.421–0.707, *p* < 0.001). Specifically, each one-point increase in the NIHSS score at 24 h was associated with a 45.5% decrease in the odds of achieving a favorable functional outcome. The presence of early signs of stroke and hemorrhagic transformation were not independently associated with 90-day functional outcome after multivariable adjustment. Although the degree of recanalization (mTICI) was significantly associated with favorable 90-day outcome in the univariate analysis, it could not be entered into the multivariable model because of complete separation: no patient with mTICI 1–2B achieved a favorable functional outcome, which precluded stable estimation of its regression coefficient.

An ordinal logistic regression analysis was performed to identify independent predictors of neurological status at discharge, with NIHSS severity categorized into three ordered levels: minor (NIHSS 1–4), moderate (NIHSS 5–15), and severe neurological deficit (NIHSS 16–42). The proportional odds assumption was satisfied and the model demonstrated good overall fit (Table 9). After adjustment for all variables included in the model, increasing age, the presence of early signs of stroke and undergoing CT perfusion imaging remained independently associated with a more severe neurological status at discharge. Specifically, each one-year increase in age was associated with a 6.3% increase in the odds of belonging to a more severe NIHSS category (OR = 1.063, 95% CI 1.008–1.122, *p* = 0.024). Patients with early signs of stroke had significantly higher odds of more severe neurological impairment at discharge compared with those without early ischemic changes (OR = 12.87, 95% CI 2.93–56.61, *p* = 0.001). Similarly, patients who underwent CT perfusion imaging had significantly higher odds of belonging to a more severe NIHSS category at discharge than those who did not undergo CT perfusion assessment (OR = 12.50, 95% CI 1.34–116.24, *p* = 0.026). The presence of in-hospital infections was not independently associated with neurological status at discharge after adjustment for the remaining covariates (OR = 1.95, 95% CI 0.46–8.65, *p* = 0.382).

Baseline, procedural, and outcome characteristics of the cohort are summarized in Table 10.

## 5. Discussion

Patients with early ischemic changes and those who underwent CT perfusion imaging had higher NIHSS scores at discharge compared with patients without early ischemic signs and those who did not undergo perfusion diagnostics. This finding most likely reflects selection bias rather than a direct effect of perfusion imaging, as CT perfusion was preferentially performed in clinically more severe or diagnostically more complex patients. A significant correlation was observed between NIHSS scores at admission and at discharge. First angiogram analysis revealed that lower mTICI score was linked with lower NIHSS at discharge. Next, appearance of infections and higher RANKIN at discharge were associated with higher NIHSS at discharge.

The presence of early ischemic signs, marked intraprocedural blood pressure variability during mechanical thrombectomy (systolic variation > 30 mmHg and/or diastolic variation > 20 mmHg), and hemorrhagic transformation were associated with higher 90-day mRS scores, whereas higher mTICI grades were associated with lower mRS scores. Prolonged duration of mechanical thrombectomy (time from groin puncture to recanalization or procedure completion) was also associated with worse 90-day functional outcomes. Lower 90-day mRS scores were observed in the case of dissection appearance and lower RANKIN at discharge, while onset of complications during the hospitalization was associated with higher 90-day mRS. In addition, advanced age and female sex were identified as negative predictors of 90-day functional outcome.

Appearance of infection early after the stoke rises risk of unfavorable outcome [11,12] while we demonstrated that onset of infection was linked with poorer NIHSS at discharge. Interestingly, NIHSS scores > 16 increased the odds for pneumonia leading to increased risk of death [13] and it has been shown that poststroke pneumonia was associated with worse functional outcome at 90 days [14]. Wang et al. demonstrated lower admission NIHSS in individuals with mTICI grade 2c/3 relative to individuals with mTICI grade 2b [15].

Despite the fact that hypertension [16,17], diabetes mellitus [18], atrial fibrillation [19], and hyperlipidemia [20] increase the risk of stroke occurrence, they did not affect either of the examined scores (the 90-day mRS or the NIHSS at discharge).

Stebner et al. analyzed the association between the 24 h NIHSS score and the 90-day mRS score and reported a strong correlation between these two measures [21]. For example, in this study we reported that in the case when presence of early stroke sings are presented, we may expect worse NIHSS at discharge and 90-day mRS; nevertheless, our results also indicated that different parameters may predict poorer outcomes depending on the scale used. For example, the presence of intraprocedural blood pressure fluctuations, defined as systolic arterial pressure changes >30 mmHg and/or diastolic arterial pressure changes >20 mmHg, was associated with worse 90-day functional outcome (mRS), but not with NIHSS scores at discharge. This further indicates that those two parameters should be examined separately, as we did in this study.

Zhang et al. pursued aims similar to ours, examining the effects of factors available at discharge on 90-day mRS outcomes. In their univariate analyses, age, hypertension, diabetes mellitus, hemorrhagic transformation, NIHSS score at discharge, and infarct volume on early follow-up imaging were significant predictors of 90-day mRS, whereas a positive history of stroke was not. In the subsequent multivariable ordinal regression model, only age and NIHSS score at discharge remained independent predictors of the 90-day mRS score [22]. In contrast to our study, their analysis did not include clinical outcome measures beyond mRS, hospital course variables (e.g., infectious complications), endovascular procedure-related parameters, or detailed radiological findings.

Gangashetty and Hegde evaluated the effects of various risk factors on 90-day mRS outcomes in 120 consecutive patients with ischemic stroke treated with intravenous thrombolysis using recombinant tissue plasminogen activator or with mechanical thrombectomy in cases of large vessel occlusion.

Their univariate analysis identified several factors significantly associated with poor 90-day outcomes (mRS 3–6): advanced age (>75 years), admission NIHSS score > 15, delayed presentation beyond 4.5 h, presence of atrial fibrillation, admission hyperglycemia > 180 mg/dL, and systolic blood pressure > 180 mmHg. Female sex approached statistical significance in its association with poor outcomes.

Multivariate logistic regression analysis revealed three independent predictors of poor 90-day functional outcome: age > 75 years, delayed presentation beyond 4.5 h, and admission NIHSS score > 15, with the latter demonstrating the strongest association with unfavorable outcomes [23].

The main advantage of our study lies in the comprehensive assessment of a broad range of parameters, including endovascular procedural performance, clinical outcomes, in-hospital course, as well as detailed clinical, radiological, and medical history data, in a homogeneous cohort of patients treated exclusively with mechanical thrombectomy.

In contrast, the majority of previous studies have evaluated a more limited set of variables in heterogeneous stroke populations treated with either mechanical thrombectomy or intravenous thrombolysis.

Compared with previous reports, the present study adds evidence in several respects. First, our cohort was treated exclusively with mechanical thrombectomy and was selected using contemporary perfusion-based imaging criteria, avoiding the heterogeneity introduced by mixing thrombectomy with intravenous thrombolysis alone. Second, clinical, radiological, procedural, and in-hospital course variables were examined jointly rather than in isolation. Third, both early neurological status (NIHSS at discharge) and long-term functional outcome (90-day mRS) were assessed in the same patients, allowing the prognostic contribution of early neurological evolution (particularly the 24 h NIHSS) to be characterized across both time horizons.

Limitations. This study has several limitations that should be considered when interpreting its findings. First, it was a single-center, retrospective analysis, which may limit the generalizability of the results and introduces the potential for selection bias inherent to this design. Second, the sample size, although adequate for the planned multivariable analyses according to the a priori power calculation, remained modest, and some associations observed in the univariate analyses may have been underpowered to reach independence in the adjusted models. Third, the single-center setting reflects the practice patterns, imaging protocols, and patient population of one tertiary stroke center, which may differ from other institutions. Despite these limitations, the study benefits from a homogeneous cohort treated exclusively with mechanical thrombectomy and from the comprehensive assessment of clinical, radiological, procedural, and in-hospital course variables. The present findings should therefore be regarded as hypothesis-generating and warrant confirmation in larger, multicenter, prospective cohorts.

## 6. Conclusions

The results of this study demonstrate that clinical, radiological, and procedural factors are significantly associated with early and long-term functional outcomes in patients treated with mechanical thrombectomy.

Recanalization success was significantly associated with long-term outcomes, with higher mTICI grades (particularly mTICI 3) associated with lower 90-day mRS scores. In contrast, marked intraprocedural blood pressure variability, hemorrhagic transformation, prolonged procedure duration, and the occurrence of in-hospital complications and infections were all associated with worse functional outcomes.

Dissection was associated with more favorable 90-day outcomes, whereas higher mRS at discharge and the development of complications during hospitalization were strong predictors of poor long-term recovery.

Overall, these findings highlight that baseline stroke severity (as assessed by admission NIHSS), successful reperfusion (defined by higher mTICI grades, particularly ≥2b/3), procedural hemodynamic stability (absence of significant intra-procedural hypotension), and prevention of in-hospital complications (especially stroke-associated pneumonia and other infections) are strongly associated with early (NIHSS at discharge) and long-term functional outcomes (90-day mRS) after mechanical thrombectomy. A multivariable binary logistic regression analysis identified only the NIHSS score at 24 h remained as an independent predictor of favorable functional outcome at 90 days.

## Figures and Tables

**Table 1:** Association of NIHSS scores at discharge and Rankin scores at 90 days with parameters of patients’ clinical status at admission and radiological findings.

Variable	NIHSS at Discharge	*p*	Rankin at 90 Days	*p*
NIHSS at admission	0.367 ^+^	0.003	0.155 ^+^	0.124
Systolic blood pressure	0.067 ^+^	0.605	0.042 ^+^	0.682
Diastolic blood pressure	−0.043 ^+^	0.742	−0.041 ^+^	0.684
CT perfusion: core volume (mL)	0.099 ^+^	0.478	0.100 ^+^	0.349
CT perfusion: penumbra volume (mL)	−0.036 ^+^	0.793	0.029 ^+^	0.784
CT perfusion: penumbra/core ratio	−0.094 ^+^	0.497	−0.085 ^+^	0.431
CT perfusion: penumbra/core mismatch ratio	−0.094 ^+^	0.497	−0.085 ^+^	0.431

^+^ Value of Spearman’s rho correlation coefficient; *p*—significance.

**Table 2:** Association of clinical and radiological characteristics of patients with NIHSS scores at discharge and Rankin scores at 90 days.

Variable		NIHSS at Discharge	Test Statistic/*p*	Rankin at 90 Days	Test Statistic/*p*
Wake-up stroke	No	6.00 (7.00)	U = 202.00; *p* = 0.464	3.00 (4.50)	U = 571.00; *p* = 0.071
	Yes	8.00 (6.00)		6.00 (4.00)	
Early signs of stroke	No	4.00 (4.75)	U = 259.50; *p* = 0.004	2.00 (4.75)	U = 750.00; *p* = 0.010
	Yes	8.00 (8.25)		5.00 (4.00)	
Hyperdense artery sign	No	6.00 (10.50)	U = 411.00; *p* = 0.771	5.00 (4.00)	U = 1034.50; *p* = 0.440
	Yes	7.00 (4.50)		3.00 (4.00)	
Occlusion, anterior circulation	Right	7.00 (7.00)	U = 459.00; *p* = 0.766	4.00 (4.00)	U = 1115.00; *p* = 0.379
	Left	6.00 (6.00)		3.00 (4.00)	
Occlusion type	Tandem	6.00 (0.0)	χ^2^ = 1.107; *p* = 0.775	4.00 (4.25)	χ^2^ = 1.694; *p* = 0.638
	ICA T	7.00 (8.50)		3.00 (5.00)	
	M1	6.00 (6.00)		4.00 (4.00)	
	M2 and M3	4.00 (11.00)		3.00 (5.00)	
CT perfusion	No	3.00 (4.00)	U = 90.50; *p* = 0.023	1.50 (5.00)	U = 317.50; *p* = 0.115
	Yes	7.00 (7.00)		4.00 (4.00)	

Results are presented as Median (Interquartile Range); χ^2^—Kruskal–Wallis test statistic; U—Mann–Whitney U test statistic; *p*—significance.

**Table 3:** Association of prior medical history characteristics and previously applied therapy with NIHSS scores at discharge and Rankin scores at 90 days.

Variable		NIHSS at Discharge	U/*p*	Rankin at 90 Days	U/*p*
Previous mRS	No symptoms	6.00 (5.50)	142.00/0.285	2.00 (2.00)	400.00/0.053
	No significant disability	6.00 (9.00)		2.00 (4.00)	
Hypertension	No	6.00 (7.00)	336.00/0.785	2.00 (1.00)	659.50/0.136
	Yes	6.00 (8.00)		2.00 (2.00)	
Atrial fibrillation	No	6.00 (5.50)	350.50/0.106	2.00 (2.00)	1050.00/0.205
	Yes	8.00 (7.50)		2.00 (1.00)	
Cardiomyopathy	No	6.00 (4.75)	337.50/0.362	2.00 (2.00)	963.00/0.601
	Yes	7.50 (7.75)		2.00 (1.50)	
Diabetes mellitus	No	6.00 (7.75)	242.50/0.737	2.00 (2.00)	654.00/0.435
	Yes	7.00 (4.75)		2.00 (2.00)	
Hyperlipidemia	No	6.00 (9.50)	269.00/0.390	2.00 (2.00)	820.00/0.743
	Yes	8.00 (4.50)		2.00 (2.00)	
Prior antiplatelet therapy	No	6.00 (6.50)	194.50/0.208	2.00 (2.00)	691.00/0.475
	Yes	7.00 (8.50)		2.00 (2.25)	
Prior anticoagulant therapy	No	6.00 (7.00)	339.00/0.824	2.00 (2.00)	865.00/0.862
	Yes	7.00 (7.00)		2.00 (1.00)	
Prior statin therapy	No	6.00 (9.00)	207.50/0.534	2.00 (2.00)	468.00/0.300
	Yes	6.00 (3.50)		1.00 (1.50)	

Results are presented as Median (Interquartile Range); U—Mann–Whitney U test statistic; *p*—significance.

**Table 4:** Association of procedural characteristics and immediate intervention outcomes with NIHSS scores at discharge and Rankin scores at 90 days.

Variable		NIHSS at Discharge	Test Statistic/*p*	Rankin at 90 Days	Test Statistic/*p*
Intervention	Mechanical thrombectomy	6.00 (7.00)	U = 58.50; *p* = 0.323	3.50 (4.00)	U = 275.00; *p* = 0.916
	Mechanical thrombectomy + thrombolysis	5.00 (/)		4.00 (4.50)	
Type of anesthesia	LA	3.50 (10.00)	χ^2^ = 5.117; *p* = 0.077	2.00 (3.50)	χ^2^ = 2.875; *p* = 0.238
	SED	4.00 (/)		1.50 (4.75)	
	OETA	7.00 (6.00)		4.00 (4.00)	
First angiogram—mTICI	0	6.00 (6.75)	U = 46.00; *p* = 0.049	3.00 (4.00)	χ^2^ = 2.481; *p* = 0.479
	1	12.00 (8.75)		4.00 (4.00)	
	2A	/		/	
	2B	/		3.50 (/)	
Stent retriever	No	6.00 (4.00)	U = 320.00; *p* = 0.438	2.00 (2.25)	U = 534.50; *p* = 0.068
	Yes	6.50 (8.50)		4.00 (4.00)	
Aspiration	No	/	/	/	/
	Yes	6.00 (7.00)		3.00 (4.00)	
Degree of recanalization (mTICI)	1	15.00 (/)	χ^2^ = 6.078; *p* = 0.108	6.00 (1.00) *	χ^2^ = 16.267; *p* = 0.003
	2A	/		/	
	2B	/		6.00 (0.50) *	
	2C	5.00 (4.25)		6.00 (4.75)	
	3	6.00 (6.00)		2.50 (4.25)	
No thrombectomy performed (7 patients)	Puncture-related problem	/	/	/	U = 1.500; *p* = 0.617
	Failed catheterization	/		4.50 (/)	
	Other	/		6.00 (0.75)	
Blood pressure variations during the procedure (SAP > 30 mmHg and/or DAP > 20 mmHg)	No	6.00 (7.25)	U = 203.50; *p* = 0.792	3.00 (4.75)	U = 561.50; *p* = 0.003
	Yes	7.00 (5.00)		6.00 (3.00)	
Intra-arterial medication during procedure	NIMO	6.00 (6.50)	/	3.00 (4.00)	/
	NIMO + IIb/IIIa	/		/	
Perioperative complications	No	6.00 (7.00)	/	3.00 (4.00)	/
	Yes	/		/	
Hemorrhagic transformation	No	6.00 (6.00)	U = 160.00; *p* = 0.115	3.00 (5.00)	U = 545.00; *p* = 0.007
	Yes	8.00 (10.00)		6.00 (3.00)	
Type of hemorrhagic transformation	HT1	7.00 (5.50)	/	3.00 (4.00)	χ^2^ = 6.113; *p* = 0.047
	HT2	/		6.00 (/)	
	PH1	/		4.50 (/)	

Results are presented as Median (Interquartile Range); χ^2^—Kruskal–Wallis test statistic; U—Mann–Whitney U test statistic; *p*—significance ; * *p* < 0.0083 compared with mTICI = 3. LA—local anesthesia; SED—conscious sedation; OETA—general endotracheal anesthesia.

**Table 5:** Association of NIHSS score at discharge and RANKIN score after 90 days with indicators of procedural characteristics and immediate intervention outcomes.

	NIHSS at Discharge	*p*	RANKIN at 90 Days	*p*
NIHSS before the start of mechanical thrombectomy	0.371	0.003	0.176	0.080
Number of passes	0.029	0.825	0.184	0.068
NIHSS after completion of the procedure	0.661	0.000	0.502	0.000
NIHSS after 24 h	0.802	0.000	0.842	0.000

Value of Spearman’s rho correlation coefficient; *p*—significance .

**Table 6:** Association of NIHSS score at discharge and RANKIN score after 90 days with time intervals related to the performance of the endovascular procedure.

Variable	NIHSS at Discharge	*p*	RANKIN at 90 Days	*p*
Time from patient entry into the stroke center (SC) to puncture	0.039	0.765	−0.015	0.885
Time from patient entry into the SC to recanalization	0.155	0.230	0.090	0.376
Duration of MT (minutes from puncture to recanalization/completion of the intervention)	0.190	0.139	0.232	0.020
Time from referral from another institution to patient entry into the SC	−0.366	0.086	−0.025	0.885
Time from notification of the neurovascular team to arrival of the on-call team	0.082	0.530	0.057	0.574

Value of Spearman’s rho correlation coefficient; *p*—significance.

**Table 7:** Association of NIHSS score at discharge and RANKIN score after 90 days with clinical outcome and hospital course.

Variable		NIHSS at Discharge	Test Statistic/*p*	RANKIN at 90 Days	Test Statistic/*p*
TOAST etiology	LAA	4.00 (5.50)	χ^2^ = 5.167; *p* = 0.160	2.00 (5.00)	χ^2^ = 2.363; *p* = 0.669
	Embolism	6.50 (9.25)		3.00 (4.00)	
	SAO	/		/	
	Other	6.00 (4.50)		6.00 (4.75)	
	Undetermined	/		/	
Dissection	No	6.50 (7.00)	U = 16.00; *p* = 0.078	4.00 (4.00)	U = 12.50; *p* = 0.030
	ICA, extracranial only	2.50 (/)		0.50 (/)	
	ICA extracranial with intracranial propagation	/		/	
Complications during hospitalization	No	6.00 (4.75)	U = 95.00; *p* = 0.081	2.00 (5.00)	U = 394.50; *p* = 0.000
	Yes	14.00 (9.00)		6.00 (1.00)	
Infections	No	6.00 (4.50)	U = 194.50; *p* = 0.031	3.00 (5.00)	U = 698.50; *p* = 0.014
	Yes	10.00 (8.50)		6.00 (3.00)	
RANKIN at discharge	0—No symptoms	/	χ^2^ = 37.650; *p* = 0.000	/	χ^2^ = 88.130; *p* = 0.000
	1—No significant disability	3.00 (4.00)		1.00 (0.00)	
	2—Mild disability	5.00 (2.00)		2.00 (1.00) *	
	3—Moderate disability	8.00 (4.25) *#		2.00 (1.00) *#	
	4—Moderately severe disability	13.00 (8.25) *#		4.00 (2.00) *#	
	5—Severe disability	14.00 (1.00) *#+		5.00 (1.00) *#+~	
	6—Death	/		/	
Death	No	6.00 (7.00)	/	2.00 (2.00)	/
	Yes	/	/	/	/

Results are presented as Median (Interquartile range); χ^2^—Kruskal–Wallis test statistic; U—Mann–Whitney U test statistic; *p*—significance; *—*p* < 0.005 compared with RANKIN at discharge = 1; #—*p* < 0.005 compared with RANKIN at discharge = 2; +—*p* < 0.005 compared with RANKIN at discharge = 3; ~—*p* < 0.005 compared with RANKIN at discharge = 4; 0.005—Bonferroni-corrected α value for post hoc comparisons.

**Table 8:** Multivariable logistic regression analysis of predictors of favorable functional outcome at 90 days (mRS 0–2).

Variable	OR	95% CI	*p* Value
Age, years	0.942	0.883–1.005	0.071
Early signs of stroke (vs. no early signs)	0.307	0.059–1.600	0.161
Hemorrhagic transformation (vs. no hemorrhagic transformation)	3.247	0.456–23.123	0.240
NIHSS score at 24 h	0.545	0.421–0.707	<0.001

Abbreviations: OR, odds ratio; CI, confidence interval. Model performance: χ^2^ = 78.431, *p* < 0.001; Nagelkerke R^2^ = 0.733; Hosmer–Lemeshow χ^2^ = 7.363, *p* = 0.498; overall classification accuracy = 86.0%. Multicollinearity diagnostics: VIF range 1.063–1.321; tolerance range 0.757–0.941.

**Table 9:** Ordinal logistic regression analysis of factors associated with NIHSS severity at discharge.

Variable	OR	95% CI	*p* Value
Age, years	1.063	1.008–1.122	0.024
Early signs of stroke (vs. no early signs)	12.87	2.93–56.61	0.001
CT perfusion performed (vs. no CT perfusion)	12.50	1.34–116.24	0.026
Infection present (vs. no infection)	1.95	0.46–8.65	0.382

Abbreviations: OR, odds ratio; CI, confidence interval. Model performance: likelihood ratio χ^2^ = 25.945, *p* < 0.001; Pearson goodness-of-fit χ^2^ = 71.984, *p* = 0.968; Deviance χ^2^ = 61.703, *p* = 0.997; Nagelkerke pseudo-R^2^ = 0.425. Proportional odds assumption: Test of Parallel Lines χ^2^ = 2.205, df = 4, *p* = 0.698.

**Table 10:** Baseline, procedural, and outcome characteristics of the study cohort (*N* = 100).

Characteristic	Value
Demographics	
Age, years	72.14 ± 11.75 (range 33–93)
Sex—male/female	37 (37%)/63 (63%)
Current smoker	49 (49%)
Clinical and radiological status at admission	
NIHSS at admission	15.35 ± 4.75 (median 16; range 4–22)
Wake-up stroke—yes/no	19 (19%)/81 (81%)
Early ischemic signs—yes/no	68 (68%)/32 (32%)
Hyperdense artery sign—yes/no	65 (65%)/35 (35%)
Systolic blood pressure, mmHg	147.60 ± 21.89 (median 145; range 100–200)
Diastolic blood pressure, mmHg	81.90 ± 11.67 (median 80; range 60–120)
ASPECTS	8.40 ± 1.13 (median 9; range 5–10)
Occlusion side—right/left	55 (55%)/45 (45%)
Occlusion type—Tandem/ICA terminus/M1/M2–M3	6 (6%)/13 (13%)/63 (63%)/18 (18%)
CT perfusion performed—yes/no	90 (90%)/10 (10%)
CTP core volume, mL	11.17 ± 12.08 (median 7; range 1–62)
CTP penumbra volume, mL	134.59 ± 65.67 (median 128; range 32–391)
CTP penumbra/core mismatch ratio	32.60 ± 37.44 (median 18; range 3–211)
Prior medical history and medication	
Pre-stroke mRS—0/1/2	77 (77%)/15 (15%)/8 (8%)
Hypertension	79 (79%)
Atrial fibrillation	43 (43%)
Cardiomyopathy	29 (29%)
Diabetes mellitus	18 (18%)
Hyperlipidemia	22 (22%)
Prior antiplatelet therapy	19 (19%)
Prior anticoagulant therapy	23 (23%)
Prior statin therapy	13 (13%)
Procedural characteristics and immediate outcomes	
Intervention—MT alone/MT + bridging IV thrombolysis	94 (94%)/6 (6%)
Anesthesia—LA/SED/OETA	5 (5%)/4 (4%)/91 (91%)
Stent retriever used	81 (81%)
Aspiration used	99 (99%)
Final mTICI—1 ^a^/2A/2B/2C/3	7 (7%)/1 (1%)/6 (6%)/12 (12%)/74 (74%)
Number of passes	1.64 ± 0.94 (median 1; range 1–5)
BP variability (SAP > 30 and/or DAP > 20 mmHg)—yes/no	24 (24%)/76 (76%)
Intra-arterial medication—nimodipine/nimodipine + IIb-IIIa	98 (98%)/2 (2%)
Perioperative complications	4 (4%)
Hemorrhagic transformation (HT1 9/HT2 11/PH1 2)	22 (22%)
Subarachnoid hemorrhage	8 (8%)
NIHSS before MT	15.41 ± 4.73 (median 16; range 4–22)
NIHSS after procedure	13.54 ± 4.69 (median 14; range 4–22)
NIHSS at 24 h	11.99 ± 5.94 (median 12; range 2–28)
Time intervals, minutes	
Stroke center entry → puncture	115.87 ± 48.67 (median 110; range 14–297)
Stroke center entry → recanalization	164.53 ± 58.56 (median 160; range 31–360)
MT duration (puncture → recanalization/completion)	48.67 ± 27.29 (median 42; range 15–145)
Hospital course and outcomes	
Length of stay, days	11.22 ± 5.98 (range 2–30)
TOAST—embolism/LAA/other/SAO/undetermined	73 (73%)/13 (13%)/12 (12%)/1 (1%)/1 (1%)
Dissection—none/ICA extracranial/ICA extra + intracranial	97 (97%)/2 (2%)/1 (1%)
In-hospital complications	23 (23%)
Infections (pneumonia)	28 (28%)
NIHSS at discharge	7.48 ± 4.57 (median 6; range 1–16)
—minor (1–4)/moderate (5–15)/severe (16–20) ^b^	18 (29%)/41 (64.5%)/4 (6.5%)
mRS at discharge—0/1/2/3/4/5/6	2%/13%/12%/19%/11%/13%/30%
mRS at 90 days—0/1/2/3/4/5/6	4%/17%/20%/9%/5%/7%/38%
In-hospital death/death by 90 days	30 (30%)/38 (38%)

^a^ Technically unsuccessful procedures (no reperfusion achieved): puncture-related problem (*n* = 1), failed catheterization (*n* = 2), other (*n* = 4). ^b^ Among survivors with available discharge NIHSS (*n* = 63). Continuous variables are presented as mean ± SD (median; range); categorical variables as *n* (%).

## Data Availability

The data presented in this study are available on request from the corresponding author due to ethics approval do not permit unrestricted public sharing of the data.

## References

[1] GBD 2021 Stroke Risk Factor Collaborators (2024). Global, regional, and national burden of stroke and its risk factors, 1990–2021: A systematic analysis for the Global Burden of Disease Study 2021. Lancet Neurol..

[2] Li X.-Y., Kong X.-M., Yang C.-H., Cheng Z.-F., Lv J.-J., Guo H., Liu X.-H. (2024). Global, regional, and national burden of ischemic stroke, 1990–2021: An analysis of data from the Global Burden of Disease Study 2021. eClinicalMedicine.

[3] Martin S.S., Aday A.W., Allen N.B., Almarzooq Z.I., Anderson C.A.M., Arora P., Avery C.L., Baker-Smith C.M., Bansal N., Beaton A.Z. (2025). 2025 Heart Disease and Stroke Statistics: A report of US and global data from the American Heart Association. Circulation.

[4] Yao M., Wang M., Ma Y., Busse J.W., Hu X., Liu Y., Luo X., Liang Q., Liang X., Zou K. (2026). Projections of the global, regional and national stroke burden by 2050: A systematic analysis for the Global Burden of Disease Study 2021. Eur. J. Epidemiol..

[5] Zhou J., Fangma Y., Chen Z., Zheng Y. (2023). Post-Stroke Neuropsychiatric Complications: Types, Pathogenesis, and Therapeutic Intervention. Aging Dis..

[6] Lucas-Noll J., Clua-Espuny J.L., Lleixà-Fortuño M., Gavaldà-Espelta E., Queralt-Tomas L., Panisello-Tafalla A., Carles-Lavila M. (2023). The costs associated with stroke care continuum: A systematic review. Health Econ. Rev..

[7] Bogicevic D., Vitosevic F., Medenica S.M., Kalousek V., Vukicevic M., Rasulic L. (2024). Direct Aspiration Thrombectomy in the Management of Procedural Thromboembolic Complications Related to Endovascular Brain Aneurysm Treatment. Medicina.

[8] Derex L., Cho T.H. (2017). Mechanical thrombectomy in acute ischemic stroke. Rev. Neurol..

[9] Lambrinos A., Schaink A.K., Dhalla I., Krings T., Casaubon L.K., Sikich N., Lum C., Bharatha A., Pereira V.M., Stotts G. (2016). Mechanical thrombectomy in acute ischemic stroke: A systematic review. Can. J. Neurol. Sci..

[10] Kwah L.K., Diong J. (2014). National Institutes of Health Stroke Scale (NIHSS). J. Physiother..

[11] Learoyd A.E., Woodhouse L., Shaw L., Sprigg N., Bereczki D., Berge E., Caso V., Christensen H., Collins R., Czlonkowska A. (2017). Infections Up to 76 Days After Stroke Increase Disability and Death. Transl. Stroke Res..

[12] Westendorp W.F., Vermeij J.D., Zock E., Hooijenga I.J., Kruyt N.D., Bosboom H.J., Kwa V.I., Weisfelt M., Remmers M.J., Houten R.T. (2015). The Preventive Antibiotics in Stroke Study (PASS): A pragmatic randomised open-label masked endpoint clinical trial. Lancet.

[13] Westendorp W.F., Dames C., Nederkoorn P.J., Meisel A. (2022). Immunodepression, Infections, and Functional Outcome in Ischemic Stroke. Stroke.

[14] Kalra L., Irshad S., Hodsoll J., Simpson M., Gulliford M., Smithard D., Patel A., Rebollo-Mesa I. (2015). Prophylactic antibiotics after acute stroke for reducing pneumonia in patients with dysphagia (STROKE-INF): A prospective, cluster-randomised, open-label. masked endpoint, controlled clinical trial. Lancet.

[15] Wang R., Huang J., Mohseni A., Hoseinyazdi M., Kotha A., Hamam O., Gudenkauf J., Heo H.Y., Nabi M., Huang J. (2024). Predictors of mTICI 2c/3 over 2b in patients successfully recanalized with mechanical thrombectomy. Ann. Clin. Transl. Neurol..

[16] Wajngarten M., Silva G.S. (2019). Hypertension and Stroke: Update on Treatment. Eur. Cardiol. Rev..

[17] Gorelick P.B., Whelton P.K., Sorond F., Carey R.M. (2020). Blood Pressure Management in Stroke. Hypertension.

[18] Menet R., Bernard M., ElAli A. (2018). Hyperlipidemia in Stroke Pathobiology and Therapy: Insights and Perspectives. Front. Physiol..

[19] Choi S.E., Sagris D., Hill A., Lip G.Y.H., Abdul-Rahim A.H. (2023). Atrial fibrillation and stroke. Expert Rev. Cardiovasc. Ther..

[20] Mosenzon O., Cheng A.Y., Rabinstein A.A., Sacco S. (2023). Diabetes and Stroke: What Are the Connections?. J. Stroke.

[21] Stebner A., Bosshart S.L., Demchuk A., Poppe A., Nogueira R., McTaggart R., Buck B., Ganesh A., Hill M., Goyal M. (2025). Factors Influencing the Association of 24-h National Institutes of Health Stroke Scale & 90-day Modified Rankin Score. Clin. Neuroradiol..

[22] Zhang M.Y., Mlynash M., Sainani K.L., Albers G.W., Lansberg M.G. (2021). Ordinal Prediction Model of 90-Day Modified Rankin Scale in Ischemic Stroke. Front. Neurol..

[23] Gangashetty T., Hegde M. (2025). Clinical Outcomes and Risk Factor Analysis in Acute Ischemic Stroke Patients: A Prospective Observational Study from a Tertiary Care Center. IOSR J. Dent. Med. Sci. (IOSR-JDMS).

